# Quantifying the impact of current and future concentrations of air pollutants on respiratory disease risk in England

**DOI:** 10.1186/s12940-017-0237-1

**Published:** 2017-03-27

**Authors:** Francesca Pannullo, Duncan Lee, Lucy Neal, Mohit Dalvi, Paul Agnew, Fiona M. O’Connor, Sabyasachi Mukhopadhyay, Sujit Sahu, Christophe Sarran

**Affiliations:** 1 School of Mathematics and Statistics, University of Glasgow, Glasgow, G12 8QW UK; 2 Met Office, FitzRoy Road, Exeter, EX1 3PB UK; 3 Met Office Hadley Centre, FitzRoy Road, Exeter, EX1 3PB UK; 4 Mathematical Sciences, University of Southampton, Highfield, Southampton, SO17 1BJ UK

**Keywords:** Air pollution, Present day and future health effects, Spatio-temporal ecological study

## Abstract

**Background:**

Estimating the long-term health impact of air pollution in a spatio-temporal ecological study requires representative concentrations of air pollutants to be constructed for each geographical unit and time period. Averaging concentrations in space and time is commonly carried out, but little is known about how robust the estimated health effects are to different aggregation functions. A second under researched question is what impact air pollution is likely to have in the future.

**Methods:**

We conducted a study for England between 2007 and 2011, investigating the relationship between respiratory hospital admissions and different pollutants: nitrogen dioxide (NO_2_); ozone (O_3_); particulate matter, the latter including particles with an aerodynamic diameter less than 2.5 micrometers (PM_2.5_), and less than 10 micrometers (PM_10_); and sulphur dioxide (SO_2_). Bayesian Poisson regression models accounting for localised spatio-temporal autocorrelation were used to estimate the relative risks (RRs) of pollution on disease risk, and for each pollutant four representative concentrations were constructed using combinations of spatial and temporal averages and maximums. The estimated RRs were then used to make projections of the numbers of likely respiratory hospital admissions in the 2050s attributable to air pollution, based on emission projections from a number of Representative Concentration Pathways (RCP).

**Results:**

NO_2_ exhibited the largest association with respiratory hospital admissions out of the pollutants considered, with estimated increased risks of between 0.9 and 1.6% for a one standard deviation increase in concentrations. In the future the projected numbers of respiratory hospital admissions attributable to NO_2_ in the 2050s are lower than present day rates under 3 Representative Concentration Pathways (RCPs): 2.6, 6.0, and 8.5, which is due to projected reductions in future NO_2_ emissions and concentrations.

**Conclusions:**

NO_2_ concentrations exhibit consistent substantial present-day health effects regardless of how a representative concentration is constructed in space and time. Thus as concentrations are predicted to remain above limits set by European Union Legislation until the 2030s in parts of urban England, it will remain a substantial health risk for some time.

**Electronic supplementary material:**

The online version of this article (doi:10.1186/s12940-017-0237-1) contains supplementary material, which is available to authorized users.

## Background

Air pollution remains a major public health problem. Despite significant improvements in air quality in western Europe and North America over the last 50 years the mortality and morbidity burden remains high. On a global scale the World Health Organisation (WHO) estimated that air pollution was responsible for the premature deaths of 3.7 million people under the age of 60 in 2012 [1]. This global problem is mirrored in the United Kingdom (UK), as it is estimated that 40,000 premature deaths are attributable to air pollution each year [2]. These health problems are likely to remain for some time, as concentrations of air pollutants are predicted to exceed limits set by European Union (EU) legislation beyond 2030 in many urban parts of the UK, especially in England (see for example [3] which relates to nitrogen dioxide). Legally established limits for air pollution do not represent thresholds below which there are no health impacts [4], and it is questionable whether thresholds for health impacts exist.

A wealth of research has been undertaken to quantify the health impact of air pollution across the world, including short-term peak concentrations [5], as well as exposure over the longer term [6]. The latter is the focus of the study described here, and cohort studies are the most popular study design for estimating such chronic effects [7]. However, such studies are expensive and time consuming to conduct, due to the need to recruit a cohort of individuals and repeatedly assess their health status for an extended follow-up period. Therefore, a relatively recent alternative uses freely available population level data relating to non-overlapping areal units for multiple consecutive years, which results in an ecological rather than an individual level study similar to that used to quantify the effects of short-term peak concentrations. Here, the effect of chronic exposure to pollution is estimated from the spatio-temporal contrasts in disease burden and air pollutant concentrations, after adjusting for population demographics and other confounders such as socio-economic deprivation. Examples of such studies in the UK include [8–12], while non-UK studies include [13–16]. Thus although these studies may be prone to ecological bias, they are used to independently corroborate the evidence from cohort studies.

There have been a number of studies that have investigated the association between air pollutants and respiratory outcomes all over the UK, where the majority of studies focus on London or England as a whole. Most recently, [17] investigated the association between long-term exposure to numerous pollutants and respiratory hospital admissions in England in 2010. They reported increased risks between 8.5 and 9.4% for a five unit increase in nitrogen dioxide (NO_2_) concentrations, and between 3.2 and 5.5% for a one unit increase in particles with an aerodynamic diameter less than 2.5 micrometers (PM_2.5_). [18] also found increased risks in respiratory hospital admissions for PM_2.5_, but not for NO_2_. Similarly, [19] observed relationships between PM_10_ and respiratory mortality, but not for NO_2_ or carbon monoxide. However, these studies only considered London, but it has been estimated that poor air quality has contributed to 4000 deaths a year. Furthermore, positive relationships have been observed in Scotland, where [12] found a 6.8% increase in cardio-respiratory mortality for NO_2_ concentrations between 2006 and 2012 in West Central Scotland, [20] found increases in respiratory hospital admissions ranging between 2.6% to 4.3% for NO_2_ and PM_2.5_ in Glasgow, and [21] found an overall 6.6% increase risk for respiratory hospital admissions for Scotland as a whole.

The aim of this paper is to present a new comprehensive study of the long-term effects of air pollution on respiratory hospital admissions in England, UK, between 2007 and 2011. In conducting this study we are motivated by two main epidemiological questions. The first is to investigate the sensitivity of the estimated pollution-health effect to the way in which representative concentrations of air pollutants are constructed at the aggregate level to align with the disease data. Air pollutant concentrations vary continuously in space and time and therefore must be aggregated in both dimensions, which for our study requires a representative concentration for each month for each Local and Unitary Authority (LUA). The average (mean) is typically used to aggregate these data [8, 10], but this risks masking periods of peak concentrations that might drive the estimated health effects. Therefore in our analysis we assess the sensitivity of our results to the choice of spatio-temporal aggregation, specifically by comparing averaging against using maximum concentrations in both space and time.

The second epidemiological question we address is what is the population-level health impact that air pollution might have in the future. We attempt to answer this difficult question by first utilising the concentrations of air pollutants and respiratory hospital admissions data between 2007 and 2011 to derive a pollution-health relationship for the present day. We then apply this estimated pollution-health relationship to future projections of climate and air quality, which allows us to make future projections of health burdens in the 2050s. In the next section we present the hospital admission, air pollutant and covariate data utilised in this study, as well as the statistical modelling that was used. The results are then presented followed by a concluding discussion.

## Methods

### Study population

The study region is England, UK (see in Additional file 1: Figure S1), with the isles of Scilly and Wight and the financial district of the City of London removed due to their small resident populations and hence very small disease counts. England had a population of around 53 million during the study period, and is partitioned into *K*=323 LUAs. Data are available for these *K* LUAs at monthly intervals between 2007 and 2011 inclusive, yielding *T*=60 consecutive time periods, making this one of the largest areal unit studies ever conducted. The disease, covariate and pollution data are described below, while additional numerical and graphical summaries are provided in Additional file 1 accompanying this paper.

### Disease data

The disease outcome data were obtained from hospital admissions records from the Health and Social Care Information Centre, where counts of the numbers of emergency hospital admissions (from all ages) in each LUA and month due to respiratory disease (International Classification of Disease 10th revision (ICD-10) codes J00-J99) were created. These counts are denoted by *Y*_*kt*_ for the *k*th LUA and *t*th month, and a summary of the counts is provided in Additional file 1: Table S1. The expected numbers of admissions *E*_*kt*_ for each month and LUA were computed using indirect standardisation, which adjusts for the varying population sizes and demographic structures across the LUA and month combinations. Specifically, $E_{kt}=\sum _{r}N_{ktr}\gamma _{r}$, where *N*_*ktr*_ is the number of people in LUA *k* during month *t* from age-sex strata *r* (e.g. males 0–5, etc), while *γ*_*r*_ is the strata specific disease rate for England.

**Fig. 1:**
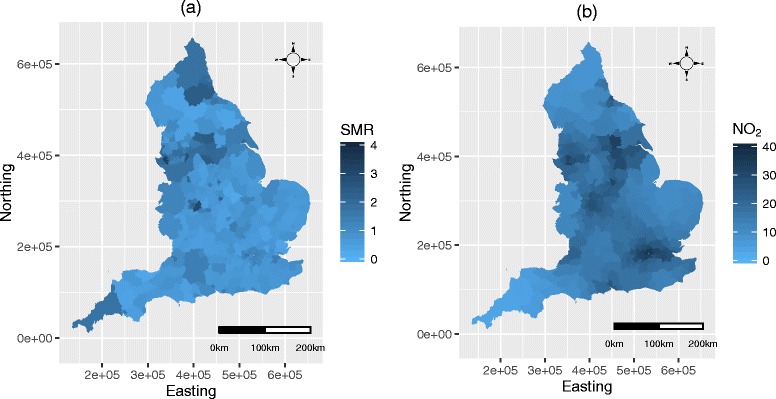
Spatial maps of respiratory SMR and NO_2_ concentrations. Panels **a** and **b** respectively display maps of the spatial pattern in the standardised morbidity ratio (SMR) for respiratory hospital admissions and NO_2_ (*μ* *gm* ^−3^) concentrations across England averaged over all *T*=60 months

The exploratory measure of disease risk is the standardised morbidity ratio (SMR) computed as SMR _*kt*_=*Y*_*kt*_/*E*_*kt*_, where an SMR of 1.2 corresponds to a 20% increased risk of disease. The map of the spatial pattern in the average SMR over all *T*=60 months is displayed in panel (a) of Fig. 1. The figure shows the highest SMRs appear around the northern cities in England, such as Leeds, Liverpool and Manchester, while the lowest SMRs appear in more rural areas. Cornwall is an exception to this generalisation, with this rural county exhibiting SMRs comparable to more urban counties. The map also exhibits localised spatial smoothness, where some pairs of neighbouring LUAs have similar values, particularly in south-east England; while other pairs of neighbouring LUAs are very different, for example in the north of England.

**Fig. 2:**
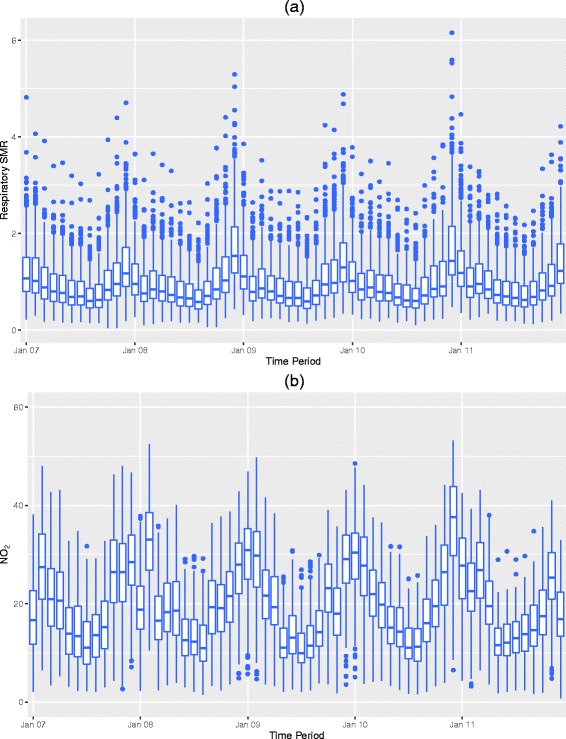
Boxplots of respiratory SMR and NO_2_ concentrations across all *T*=60 months. Panels **a** and **b** respectively display boxplots of the temporal pattern in the standardised morbidity ratio (SMR) for respiratory hospital admissions and NO_2_ (*μ* *gm* ^−3^) concentrations across all *T*=60 months

The temporal pattern in disease risk is displayed in panel (a) of Fig. 2, which shows a clear seasonal pattern across the years, where higher respiratory risks are present in the winter months due to colder temperatures leading to increases in influenza cases. However, heatwaves (in summer) can also have a negative impact on human health, for example, the 2003 summer heatwave had tens of thousands of attributed deaths [22]. Furthermore, the occurrence of such heatwaves are likely to increase in the future due to the increasing effects of climate change [23]. We therefore adjust for the seasonality in the SMR in two ways. Firstly, we apply a monthly correction factor to *E*_*kt*_ to make it seasonal, and secondly we include a measure of temperature as a covariate in the model (see covariate section below). Finally, the seasonally-adjusted SMR does not vary greatly across the 5-year period as evidenced by the similar distributions for each year (see Fig. 2).

### Pollution data

Concentrations of air pollutants across the UK are measured by the Department for the Environment, Food and Rural Affairs (DEFRA) Automatic Urban and Rural Network (AURN), and in addition to this network local authorities also monitor selected pollutants in key locations where levels are elevated. However, air pollution is often highly spatially heterogeneous, and these measured observations are sparse at the LUA level, with some LUAs having no observations. In order to derive air pollutant concentrations at all points across the UK it is therefore essential to use an air quality model.

Our study uses present-day and future projections of air pollutant concentrations derived from configurations of the UK Met Office’s Unified Model (MetUM) [24]. Present-day concentrations for the UK were generated by the Met Office operational air quality forecast model AQUM [25], operated in a hindcast mode. The raw model hourly output data were combined with corresponding hourly surface air pollution measurements by the technique described in [26], to produce improved estimates of pollutant concentrations over the whole UK. The model operates at a spatial resolution of 12km and does not explicitly resolve the fine structure of emissions in urban areas. However in view of the spatial and temporal averaging employed in this study, relating pollutant data to health data, this does not present a serious limitation. The future climate and air quality projections were generated by AQUM, nested within regional and global climate-composition models and are fully discussed in (Folberth, GA: Future projections of UK air quality and implications for health, in preperation). Other examples of health studies using modelled pollution data include [10, 27–29].

The air quality model provides hourly concentrations for five pollutants: nitrogen dioxide (NO_2_); ozone (O_3_); particulate matter, the latter including particles with an aerodynamic diameter less than 2.5 micrometers (PM_2.5_), and less than 10 micrometers (PM_10_); and sulphur dioxide (SO_2_). For each pollutant, daily mean and daily maximum concentrations were calculated from the hourly concentrations, which were then both aggregated to the monthly level by taking the arithmetic mean across the days in each month. These monthly concentrations are spatially misaligned to the disease data, since they relate to the 12km model grid and not to the irregularly-shaped LUA boundaries. We rectify this by computing both a spatial mean (a mean of the grid boxes that fall within the LUA) and a spatial maximum (the maximum of the grid boxes that fall within theLUA) for each LUA and pollutant. Therefore, to assess the robustness of our conclusions to the choice of pollutant aggregation function, the following 4 metrics are computed for each pollutant:

- spatial mean of the temporal mean (*m* *e* *a* *n*_*s*_.*m* *e* *a* *n*_*t*_);
- spatial mean of the temporal maximum (*m* *e* *a* *n*_*s*_.*m* *a* *x*_*t*_);
- spatial maximum of the temporal mean (*m* *a* *x*_*s*_.*m* *e* *a* *n*_*t*_);
- and spatial maximum of the temporal maximum (*m* *a* *x*_*s*_.*m* *a* *x*_*t*_).

In the above the subscript _*s*_ denotes spatial and subscript _*t*_ denotes temporal. The use of these metrics allow us to assess whether peak concentrations over time and space have a stronger association with respiratory disease compared to average concentrations.

Figures and Tables summarising the average concentrations and correlations between the present-day aggregated pollutant metrics are displayed in Additional file 1 accompanying this paper. However, panel (b) in Fig. 1 displays the spatial pattern in the NO_2_ *m* *e* *a* *n*_*s*_.*m* *e* *a* *n*_*t*_ metric averaged across all time periods, while panel (b) in Fig. 2 displays its temporal pattern via boxplots for each month. The latter highlights a clear seasonal pattern in which NO_2_ concentrations peak in the colder months, while no trend is seen over the 5 years. The map in Fig. 1 (b) highlights that spatial peaks in NO_2_ occur around large cities, such as Birmingham.

Future projections of climate and air quality for the 2050s were produced using the same nested configuration of MetUM, but using greenhouse gas concentrations, aerosol and aerosol precursor emissions, and tropospheric ozone (O_3_) precursor emissions for the 2050s following three of the Intergovernmental Panel on Climate Change (IPCC) fifth assessment report’s (AR5) Representative Concentration Pathways (RCPs): RCP2.6 [30], RCP6.0 [31], and RCP8.5 [32]. Corresponding sea surface temperatures (SSTs) and sea ice (SI) conditions were taken from simulations of the HadGEM2-ES model [33] run with the same climate forcings and air quality emissions [34]. While these pathways capture the possible range of future climate in the 2050s, they uniformly assume the global implementation of air quality policies, with aggressive reduction of air pollutant emissions in the 2050s relative to the present-day, with the exception of methane (CH_4_) in RCP8.5, an important precursor of tropospheric O_3_. As a result, they do not capture the full range of possible future air quality [35]. Future projections were produced for all aforementioned pollutants on the same 12km square grid under each of the three RCPs. As with the present day concentrations, daily means and daily maximum concentrations were computed from the hourly data, which were then aggregated to the monthly and LUA level using the same metrics as the present-day pollution data. These data are again summarised in Additional file 1.

### Covariate data

One of the key confounding factors in ecological pollution-health studies is socio-economic deprivation [36], since populations with higher levels of socio-economic deprivation are more likely to undertake risky behaviours such as smoking and poor diet, and thus have poorer respiratory health overall. Socio-economic deprivation is a combination of multiple factors which makes it difficult to measure, and so we use two proxy measures, namely: the percentage of the working age population that is in receipt of Job Seekers Allowance (JSA) (a benefit paid to working age people out of work), and the median property price (MPP). JSA and MPP are available for all LUAs across England, but only at the annual temporal resolution, and the distributions are summarised in Additional file 1: Table S1 accompanying this paper. It was not possible to assess whether smoking was a confounder in our analyses as information on smoking was not available at the LUA level. Smoking should be highly correlated with deprivation, which we have adjusted for. This was demonstrated in the paper by [18] who showed that in London at the borough level smoking prevalence was linearly related to JSA (Pearson’s correlation coefficient of 0.67) suggesting that deprivation variables, such as JSA, can serve as a proxy variable for smoking. In addition, temperature is well known to impact respiratory disease [37], with very cold and very warm temperatures leading to increased admissions. For this study we use the average modelled temperature in each LUA and month, and again these data are summarised in Additional file 1: Table S1.

### Statistical analysis

Poisson log-linear models are typically used to model these data, where the spatio-temporal pattern in disease risk is modelled by known covariates and spatio-temporally autocorrelated random effects [10, 18]. The latter are included in the model to account for residual spatio-temporal autocorrelation, which is autocorrelation remaining in the disease data after adjusting for known covariates. This autocorrelation is caused by many factors, such as unmeasured confounding, neighbourhood effects (where the behaviour of individuals is influenced by the behaviour of neighbouring individuals), grouping effects (where individuals choose to be close to similar individuals), and the fact that successive observations in the same unit relate to largely the same susceptible population. The presence of this residual autocorrelation violates the assumption of independence made in simple regression models, requiring the inclusion of autocorrelated random effects in the model.

Typically, a Gaussian Markov Random Field (GMRF) model is specified for these random effects [8–10, 13–16, 18, 20], and inference for the model is set in a Bayesian setting using Markov chain Monte Carlo (MCMC) simulation. The model used here was proposed by [38], and has the general form:

1 $$\begin{array}{@{}rcl@{}}  Y_{kt} | E_{kt}, R_{kt} &\sim& \text{Poisson}(E_{kt}R_{kt}), \\ \ln(R_{kt}) &=& \mathbf{x}_{kt}^{\top}\boldsymbol{\beta} + \phi_{kt}, \end{array} $$

where *R*_*kt*_ denotes the overall disease risk in the *k*th LUA and *t*th month relative to the expected disease count *E*_*kt*_. The vector of known covariates (air pollutant, socio-economic deprivation and temperature) is denoted by **x**_*kt*_, while the corresponding regression parameters are denoted by ***β***. The spatio-temporal random effect for area *k* and month *t* is denoted by *ϕ*_*kt*_, while the vector for all LUA for month *t* is denoted by ***ϕ***_*t*_=(*ϕ*_1*t*_,…,*ϕ*_*Kt*_). The GMRF prior used here models these effects as:

2 $$\begin{array}{@{}rcl@{}} \boldsymbol{\phi}_{t} | \boldsymbol{\phi}_{t-1} &\sim& \mathrm{N}(\gamma \boldsymbol{\phi}_{t-1}, \tau^{2} \mathbf{Q}(\mathbf{W},\rho)^{-1}),\; t = 2, \ldots, T,\\ \boldsymbol{\phi}_{1} &\sim& \mathrm{N}(\mathbf{0}, \tau^{2} \mathbf{Q}(\mathbf{W},\rho)^{-1}), \end{array} $$

which is a multivariate first order autoregressive process. Temporal autocorrelation is modelled via the mean *γ* ***ϕ***_*t*−1_, where *γ* is the temporal autocorrelation parameter, with a value of zero corresponding to independence while a value of one corresponds to strong temporal autocorrelation. The spatial structure in the data is represented by **W**, a binary *K*×*K* spatial adjacency matrix, where *w*_*kj*_=1 if the (*k*,*j*)th LUAs share a common border and is zero otherwise (diagonal elements *w*_*kk*_=0). Thus, spatial autocorrelation in the data is modelled by the singular precision matrix

3 $$ \mathbf{Q}(\mathbf{W},\rho) = \rho\,[\!\text{diag}(\mathbf{W}\mathbf{1}) - \mathbf{W}] + (1-\rho)\mathbf{I},  $$

where **1** is a *K*×1 unit vector and **I** is the *K*×*K* identity matrix. This precision matrix corresponds to the Gaussian Markov Random Field prior proposed by [39], and is commonly used for spatial areal unit modelling applications. Here *ρ* is the spatial dependence parameter, with a value of zero corresponding to independence while a value of one corresponds to strong spatial autocorrelation. However, this model captures globally smooth spatial autocorrelation, which as shown in Additional file 1: Figure S4 is not suitable for our data. The figure shows a spatial map of the residuals on the log-scale (averaged over time) from a simplified model including only the covariates (the random effects are removed), from which localised spatial smoothness that is present between some pairs of neighbouring areas but absent between others is clearly visible.

Thus here we treat the elements of **W** corresponding to pairs of spatially adjacent LUA as random quantities to be estimated, rather than being fixed at one. If the corresponding *w*_*kj*_ element is estimated as close to one then strong spatial autocorrelation is assumed between (*ϕ*_*kt*_,*ϕ*_*jt*_) for all time periods *t*, while if it is estimated as close to zero then the random effects are modelled as conditionally independent. Full details of this model are given by [38], and the model can be implemented using the CARBayesST software in the statistical software R.

We quantify the impact of future air pollution on respiratory hospitalisation rates by estimating the change in the numbers of hospital admissions that would occur if the present-day air pollutant concentrations from 2007 to 2011 were replaced by the future projections. The first step to achieving this is to compute the relative risk of hospitalisation comparing the current and future air pollutant concentrations. That is for area *k* and year *t* (e.g., comparing January 2007 against January 2050, February 2008 against February 2051, etc) we have

4 $$\begin{array}{*{20}l} \text{RR}_{kt} &= \frac{\text{Expected number of admissions given current levels}}{\text{Expected number of admissions given projected levels}}\\ &=\frac{E_{kt}\exp\left(\mathbf{x}_{kt}^{\top}\hat{\boldsymbol{\beta}} + \phi_{kt}\right)}{E_{kt} \exp\left(\mathbf{z}_{kt}^{\top}\hat{\boldsymbol{\beta}} + \phi_{kt}\right)}\\ &= \exp\left(\left[ z_{kt}^{(p)} - x_{kt}^{(p)}\right] \hat{\beta}^{(p)} \right). \end{array} $$

In the above equation (**x**_*kt*_,**z**_*kt*_) respectively denote the vector of covariates for the present-day and future, and only differ in their pollutant concentrations $(x_{kt}^{(p)}, z_{kt}^{(p)})$. The remaining socio-economic deprivation and temperature covariates relate to the present day values. Finally, $\hat {\beta }^{(p)}$ is the estimated relationship between air pollution and disease risk from the present-day analysis. Then, based on the present-day population, if pollution over the 5-year period changed from the 2007–2011 levels to the 2050s levels then the estimated annual average number of increased / reduced admissions over all *K*=323 LUA would be:

5 $$ \hat{n} =\frac{1}{5} \sum_{k=1}^{323}\sum_{t=1}^{60}RR_{kt}Y_{kt} - Y_{kt}.  $$

The first term *R* *R*_*kt*_ *Y*_*kt*_ is the estimated number of respiratory hospital admissions if the present-day pollution levels were replaced by the future projections, while the second term is obviously the number of admissions resulting from the present-day air pollutant concentrations. For example, if the concentrations had not changed, that is if $z_{kt}^{(p)} = x_{kt}^{(p)}$ then the relative risk *R* *R*_*kt*_=1 and $\hat {n}=0$ as would be expected.

## Results

The aforementioned statistical model is fitted in a Bayesian setting via MCMC simulation using the CARBayesST package for R available from https://cran.r-project.org. All the results presented are based on 10,000 MCMC samples, that were generated by running a Markov chain for 120,000 samples of which the first 20,000 were discarded as the burn-in period (by which point convergence was assessed to have been reached) and the remaining 100,000 were thinned by a factor of 10, by retaining every 10th data point, to reduce their autocorrelation.

Each aggregation metric for each pollutant was included in a separate disease model due to the collinearity between the different pollutants and between the different aggregation metrics for the same pollutant. The covariates in each model included a single measure of pollution, temperature and socio-economic deprivation, the latter including the percentage of the working age population in receipt of job seekers allowance and the median property price in each LUA.

Fitting the models showed there was strong spatio-temporal autocorrelation present in the disease data after adjusting for the known covariates, since the estimated spatial and temporal autocorrelation parameters were $(\hat {\rho }=0.98, \hat {\gamma }=0.99)$. Values of zero for these parameters correspond to independence, while values close to one correspond to strong autocorrelation.

The estimated relative risk for temperature for a 4.682 °C (equating to one standard deviation) increase ranged between 0.867 and 0.922 depending on the pollution metric included in the model, which is around a 10% reduction in risk for a 4.682 °C increase. These estimated effects were significant at the 5% level, as the 95% credible intervals (the Bayesian equivalent of frequentist confidence intervals) were wholly below the null risk of one. This negative relationship with the risk of respiratory hospital admissions is somewhat expected, since colder temperatures are known to result in greater numbers of hospital admissions. However, the possibility of a non-linear relationship was considered for temperature to account for the potential harmful effects of heatwaves, but exploratory analyses suggested a linear relationship was sufficient for the data.

**Fig. 3:**
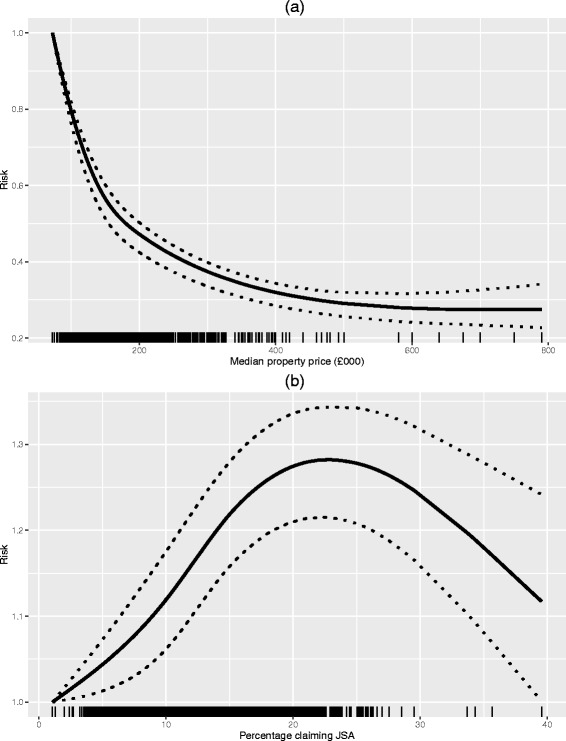
Estimated risks between MPP and JSA against the risk of respiratory admission. Panels **a** and **b** respectively display the estimated non-linear relationships between the risk of respiratory hospital admission and the two deprivation covariates, namely median property price and job seekers allowance claimants. The solid curve denotes the estimated relationship, and the dashed lines denote the 95% credible intervals. The tick marks on the x axis represent the locations of the data points in covariate space

Both the socio-economic deprivation covariates JSA and MPP were allowed to exhibit non-linear relationships with disease risk, which was achieved by modelling their effects with natural cubic splines with 3 degrees of freedom. The resulting relationships are displayed in Fig. 3, where the top plot refers to MPP, and the bottom plot refers to JSA. In both cases the tick marks on the x axis relate to the values of each covariate in the data set. The figure shows that both covariates suggest that disease risk increases as socio-deprivation increases, which relates to a decrease in MPP and an increase in JSA. The apparent reduction in risk as JSA increases from 20 to 40% is likely to be spurious, as it is based on few data points (see the tick marks on the x axis) and a horizontal line (representing no change) fits between the wide 95% credible intervals. Finally, both covariates show strong evidence of non-linear behaviour on disease risk, as straight lines cannot be drawn to stay within the 95% credible intervals across the entire range of the covariates.

The estimated relationships between each pollutant and aggregation metric and respiratory hospital admissions are displayed in Table 1, where all results are presented as relative risks for a one standard deviation increase in each metrics value. These standard deviation increases are presented in the table caption. The table shows that NO_2_ associated with respiratory hospital admissions across all aggregation metrics, since the relative risks are all positive and their 95% credible intervals are wholly above the null risk of one. The aggregation metric with the highest relative risk of 1.016 was for the *m* *e* *a* *n*_*s*_.*m* *e* *a* *n*_*t*_ metric, indicating that for around a 10 *μ* *g* *m* ^−3^ increase in NO_2_ the risk of respiratory hospital admissions increases by 1.6%. The magnitude of the differences in the estimated effects across the four aggregation metrics is small in absolute size, as the estimates range between 1.009 and 1.016, suggesting that these results are relatively robust to the choice of a representative aggregated measure of pollution.

**Table 1:** Posterior medians and 95% credible intervals (in brackets) for the estimated relationship between each pollutant and aggregation metric and respiratory hospitalisation

Pollutant	mean _*s*_.mean _*t*_	mean _*s*_.max _*t*_	max _*s*_.mean _*t*_	max _*s*_.max _*t*_
NO_2_	1.016 (1.008, 1.028)	1.014 (1.009, 1.011)	1.009 (1.000, 1.016)	1.011 (1.004, 1.016)
O_3_	0.985 (0.973, 0.995)	0.992 (0.980, 1.002)	0.986 (0.974, 0.997)	0.998 (0.986, 1.008)
PM_10_	1.008 (0.999, 1.020)	1.004 (0.994, 1.012)	1.006 (0.994, 1.022)	1.005 (0.993, 1.014)
PM_2.5_	1.008 (0.997, 1.024)	1.006 (0.996, 1.013)	1.010 (0.999, 1.020)	1.004 (0.997, 1.014)
SO_2_	1.000 (0.995, 1.004)	0.999 (0.995, 1.005)	0.999 (0.994, 1.004)	0.999 (0.995, 1.005)

Results are presented as relative risks for a 1 standard deviation increase in each pollutants value (measured in *μ* *gm* ^−3^) which are (in the order of the aggregation metrics below): NO_2_ (9.56, 13.63, 9.6, 13.51), O_3_ (13.55, 15.8, 13.79, 15.97), PM_10_ (5.1, 7.47, 5.17, 7.52), PM_2.5_ (4.22, 6.05, 4.22, 6.06), SO_2_ (1.38, 3.48, 1.77, 3.83)

PM_10_ and PM_2.5_ are estimated to have marginal impacts on increased respiratory hospitalisation, since their relative risks are all above one for each aggregation metric. PM_2.5_ exhibits slightly higher estimated risks compared to PM_10_. However, for both pollutants the estimated effect sizes are closer to one compared with NO_2_ and are not substantial at the 5% level, the latter being because the 95% credible intervals include the null risk of one.

SO_2_ exhibits no relationship with respiratory hospital admission risk for any of the aggregation metrics, with all estimated relative risks being equal to one to two decimal places. In contrast, O_3_ showed a negative association with the risk of respiratory hospital admission, that is increasing concentrations being estimated to reduce respiratory admissions. However, this is likely due to confounding from the study design, because O_3_ has a negative correlation with NO_2_ (-0.68 for the *m* *e* *a* *n*_*s*_.*m* *e* *a* *n*_*t*_ metric). This negative correlation means that as NO_2_ has a positive effect then a negatively correlated pollutant such as O_3_ will likely have an estimated effect of the opposite sign.

Sensitivity analyses were performed investigating the effect of two-pollutant models in order to take into account the contemporaneous effects of two pollutants on the risk of respiratory hospital admissions. The correlation between the pollutants were relatively high, however the correlation between NO_2_ and PM_2.5_ was only 0.540, and -0.68 between NO_2_ and O_3_. The Committee on the Medical Effects of Air Pollutants [40] suggest that associations with O_3_ could be masked when there is no adjustment for negatively correlated pollutants, such as NO_2_. Utilising the aforementioned methodology two extra models were considered looking at the joint effects of NO_2_ with PM_2.5_, and NO_2_ with O_3_ for the *m* *e* *a* *n*_*s*_.*m* *e* *a* *n*_*t*_ metric. The relative risks for the NO_2_-PM_2.5_ model are as follows: 1.014 (1.008, 1.028) for NO_2_, and 1.003 (0.990, 1.015) for PM_2.5_. The relative risks for the NO_2_-O_3_ model are as follows: 1.015 (1.002, 1.029) for NO_2_, and 1.001 (0.980, 1.015) for O_3_. These effects show slight attenuation for NO_2_ and PM_2.5_ compared to the single-pollutant only models (see Table 1) highlighting the robustness of these effects when only considered on their own. For O_3_, the effect changes from slightly negative (with a credible interval close to the null risk of one) to no association, while NO_2_ only shows slightly attenuation. Again, this highlights the robustness of our findings when only single-pollutant models are considered. In addition, it has been demonstrated that hot and cold temperatures can affect the respiratory system, therefore monthly minimum and maximum temperatures were calculated and included in a model with the NO_2_ *m* *e* *a* *n*_*s*_.*m* *e* *a* *n*_*t*_ metric. The correlation between average and minimum temperature was 0.973, and 0.948 between average and maximum temperature. The relative risks for NO_2_ increased to 1.018 (1.012, 1.028) and 1.017 (1.006, 1.026) for maximum and minimum temperature respectively, compared to a relative risk of 1.016 (1.008, 1.028) when average temperature was used. These results suggest that due to the high correlation between the temperature metrics the relative risk for NO_2_ is consistent. The relative risk for temperature is consistent across the three measures, ranging from 0.892 (0.858, 0.919) for average temperature, 0.962 (0.921, 0.979) for minimum temperature, and 0.989 (0.965, 0.999) for maximum temperature. However, the effects for temperature are slightly attenuated when minimum and maximum values are used. Therefore, in our study the effect of temperature on respiratory hospital admissions is consistent across the three measures. In addition to the sensitivity analyses conducted above, another model was performed that investigated the joint effect of PM_10_ and O_3_ since the correlation between these two pollutants was extremely low at -0.083. For the *m* *e* *a* *n*_*s*_.*m* *e* *a* *n*_*t*_ metric the relative risk for PM_10_ was 1.001 (0.987, 1.011), and 0.985 (0.975, 1.000) for O_3_. The effect for O_3_ did not change compared to the single pollutant model, and there was slight attenuation for the PM_10_-health effect. Likewise, these results emphasise the robustness of the effects when only considered in single pollutant models.

In the above results NO_2_ exhibited the strongest effect on respiratory hospital admissions since its 95% credible intervals did not contain the null risk of one, and is thus chosen to estimate the impact of future climate and air quality on the future risk of hospitalisation due to respiratory disease. In this analysis the results for the *m* *e* *a* *n*_*s*_.*m* *a* *x*_*t*_ metric are presented in Table 2, while the results for the other metrics are similar and are discussed in the supplementary material. The method outlined in the previous section is used, whereby we first compute the relative risk using (4), then estimate the yearly average number of attributable respiratory hospital admissions using (5). In RCPs 2.6, 6.0, and 8.5, the number of respiratory hospital admissions in England are projected to decrease in the 2050s by 10,478, 8,659, and 14,661, respectively. The average number of present-day respiratory hospital admissions per year is 613,052 across England, thus these decreases relate to RCP2.6 - 1.7%, RCP6.0 -1.4%, and RCP8.5 - 2.4% reductions compared with the current admissions rates. Thus the estimated numbers of admissions reduce across all three pathways, with climate scenario RCP8.5 producing the highest reduction and RCP6.0 producing the lowest. These projected decreases in respiratory admissions are driven by projected decreases in NO_2_ concentrations across all 3 RCPs. It is important to note that a RCP which has a higher mean temperature increase does not necessarily imply higher air pollutant concentrations.

**Table 2:** Projected NO _*x*_ emission totals under the three RCPs and the percentage of the present-day (2007–11) emission totals for UK-only emissions that each one relates to

RCP	Total emissions	% of present-day	Number of reduced
	(kg/s)		admissions $\hat {n}$
2.6	21.46	69.23	10,478 (15,435, 4,367)
6.0	23.25	75.02	8,659 (12,740, 3,615)
8.5	13.22	42.66	14,661 (21,546, 6,128)

The total present-day NO _*x*_ emissions are 30.99 kg/s. Also presented are the estimated reductions in respiratory admissions per year and 95% credible intervals in brackets

To interpret our results one must consider how the nitrogen oxide (NO _*x*_ = NO and NO_2_) emissions vary between the three RCPs. The average projected NO _*x*_ emissions (kg per second) for the UK for each RCP for the 2050s is displayed in Table 2, which also displays the emissions as a percentage of present day values. The table displays reductions of between 42.66 and 75.02% under each RCP compared to present-day NO _*x*_ emission totals. Therefore, it is clear that NO _*x*_ emissions significantly reduce under the future climate projections. This is the main driver for the reductions in projected hospital admissions due to respiratory disease in all RCPs. These projected reductions in admissions are also displayed in Table 2, and range between 8659 and 14,661 per year. RCP6.0 has the lowest reduction in projected respiratory hospital admissions compared to the other two scenarios, consistent with the fact that it also has the smallest reduction in NO _*x*_ emissions. Variations also arise amongst the different RCPs due to emissions of other pollutants (for example, SO_2_ has the highest emissions in RCP6.0), non-linear chemistry feedbacks in the atmosphere, and the differing meteorology, which in turn all affect air concentrations of NO_2_.

**Fig. 4:**
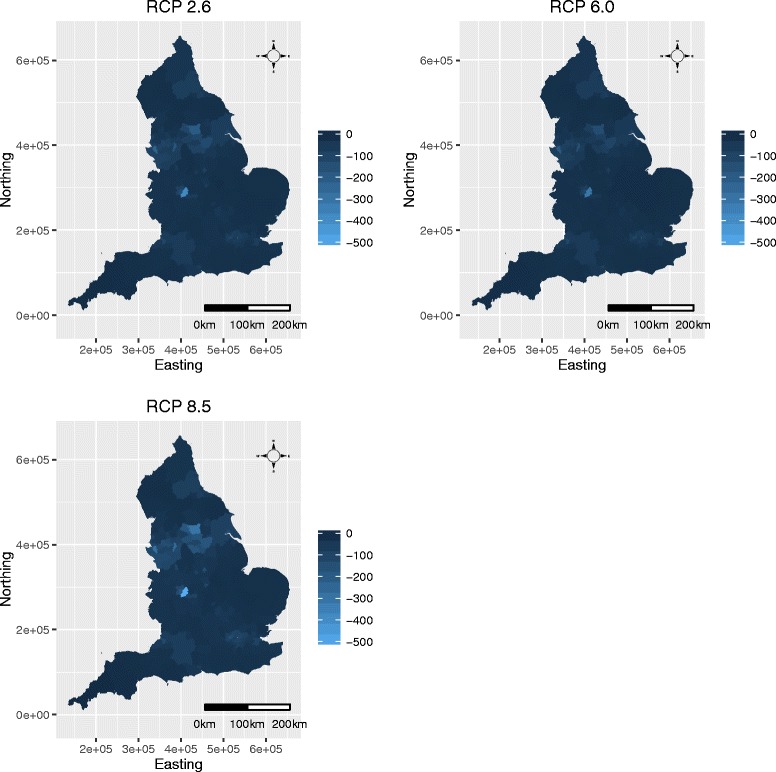
Spatial maps displaying the projected yearly numbers of reduced hospital admissions. The 3 maps show the projected yearly average decreases in the numbers of respiratory hosptialisations based on the 2050-2054 NO_2_ concentrations based on each of the three RCPs (2.6, 6.0, 8.5)

Figure 4 displays the spatial distribution of the projected yearly average reductions in respiratory hospitalisations by LUA from all 3 RCPs across England. In the figure the minus numbers denote the size of the projected reductions in hospital admissions if the current day *m* *e* *a* *n*_*s*_.*m* *a* *x*_*t*_ NO_2_ concentrations were replaced by the projections under RCPs 2.6, 6.0 and 8.5. The maps indicate that in all 3 RCPs the highest reductions in NO_2_ concentrations and hence respiratory admissions occur in the urban cities of London (south east), Birmingham (central) and Liverpool, Manchester and Leeds (north). In contrast, there are smaller changes in the more rural areas.

## Discussion

This paper has presented a new comprehensive study of the long-term effects of air pollution on respiratory hospitalisation rates in England between 2007 and 2011, as well as estimating the future impact of air pollution for the 2050s. The study is at the LUA and monthly resolution, and with 19,380 spatio-temporal observations is one of the largest spatio-temporal areal unit studies ever conducted. It was also straightforward to implement, making use of routinely available aggregated respiratory hospital admissions records and modelled regional air pollutant concentrations. Both these data sets are available in Additional file 2 accompanying this paper, and the sophisticated spatio-temporal models used to estimate the pollution-health effects are freely available via the R package CARBayesST. The two key aims of this study were to: (i) examine the sensitivity of the estimated pollution-health effect to the approach taken to computing a representative measure of air pollutant concentrations for each LUA and month; and (ii) use the estimated pollution-health relationship and future projections of concentrations to estimate the potential health burden from air pollution in the future.

In regard to aim (i), a representative LUA and monthly air pollutant concentration is typically computed by averaging concentrations over space and time. In this paper we compared four different aggregation metrics based on computing averages and maximums in space and time, and our main finding is that the results showed very little sensitivity to the choice of metric. The consistency of the results across aggregation metrics is a reassuring finding, and suggests, for this study at least, that the choice of aggregation function did not impact the substantive conclusions. However, it is slightly at odds with the results from [41] in Scotland, who found that the spatial maximum NO_2_ concentrations produced significant health effects, while the spatial mean had no such relationship. Therefore in future it would be interesting to conduct similar sensitivity analyses in studies at different locations to assess whether the robustness observed here holds more widely.

For all aggregation metrics NO_2_ exhibited clear associations with respiratory hospital admissions since the 95% credible intervals were wholly above the null risk of one, with estimated increased risks of between 0.9 and 1.6% for a one standard deviation increase in concentrations. This result suggests that, as NO_2_ concentrations are predicted to exceed EU legislation in parts of urban England until at least the 2030s [3], NO_2_ will be an ongoing health concern for some time. In contrast, the two particulate matter metrics exhibited much smaller (non-significant) but still positive associations with respiratory hospital admissions, while SO_2_ exhibited no association at all. Furthermore, the pollutants were included separately in the models due to their relatively high pairwise correlations. It has been shown that combining NO_2_ and O_3_ into a single metric produces pollution-health associations of a greater magnitude compared to conducting two single-pollutant analyses [42]. However, in our sensitivity analysis we ran a model with both NO_2_ and O_3_ as covariates, where the effect sizes were only slightly attenuated. Therefore, future work could investigate this relationship within this spatial ecological setting to see whether similar results are found when combing into one NO_2_-O_3_ metric. The relationships observed here are in line with previous ecological studies conducted in England where [17] found stronger effects on respiratory hospital admissions for NO_2_ compared to PM_2.5_ and PM_10_. They estimated stronger effects for NO_2_ of between 8.5 and 9.4% compared to our study where the effects ranged between 0.9 and 1.6%. However, this was a purely spatial study conducted in 2010 and only average temporal and spatial aggregated metrics were considered. Information on smoking prevalence was not available, however the same deprivation measures were used and are thus sufficient in the control for smoking. [18] conducted a spatio-temporal study in London where higher relative risks in respiratory hospital admissions were observed for PM_2.5_ compared to our study (1.8% versus 0.8%). In contrast, they found no association for NO_2_ concentrations since its credible interval contained the null risk of one, but the estimated relative risk of 1.013 is in line with our observed relative risk of 1.016 for NO_2_. Again, no information on smoking was available at their chosen area level, however the authors state that their deprivation measures were sufficient at acting as a proxy variable for smoking. Stronger effects were observed for respiratory mortality in the study conducted by [9] across electoral wards in Great Britain, where concentrations of black smoke produced an excess risk of 3.6% (2.6%, 4.5%). However, only wards that contained a monitoring site were included, meaning many wards and thus important information had to be discarded. This highlights the need for spatially complete air pollutant data so that all available information can be utilised. Finally, our results also align with studies conducted in Scotland, where stronger risks are observed for NO_2_ compared to other pollutants [10, 12, 20, 41, 43]. Furthermore, [41] computed the spatial maximum NO_2_ concentrations as well as the typical average aggregation metric, where they found increased associations with the spatial maximum rather than with the spatial mean. Conversely, we found slightly stronger effects when the spatial mean was used rather than the spatial maximum, therefore more work is needed in order to understand this phenomenon further.

The results addressing the second aim of this study suggest that in the future the impact of NO_2_ concentration on respiratory hospital admissions will fall, with estimated decreases in admissions of between 1.4% and 2.4% depending on which of the 3 RCPs used here are considered. However, the RCPs considered all assume a reduction in NO _*x*_ emissions across the UK, which is a partial driver of projected falls in NO_2_ concentrations. Therefore it would be interesting in future work to compare results from the new range of climate and air quality scenarios being developed for the next IPCC assessment [44], which will consider different air quality policies for a given climate change scenario.

From a methodological perspective, the statistical models assumed in this paper, and in almost all the existing literature, assume the aggregated pollutant concentration for each areal unit and time period is fixed and known precisely, whereas in fact it is an error prone estimate. Therefore the uncertainty in its value should be accounted for when estimating its health effects. One approach to achieving this was proposed by [45], who created an exposure distribution for each areal unit and time period, and averaged the estimated pollution-health effects over this exposure distribution. However, a number of approaches are possible for accounting for this exposure uncertainty, and a rigorous comparison of the different approaches is much needed.

## Conclusions

In conclusion, NO_2_ concentrations exhibit the greatest association with respiratory hospital admissions in our study. This result is consistent across the different aggregation metrics used in the analysis. In view of the continuing, relatively high concentrations predicted for the next 15 or so years [3], this pollutant is likely to remain a serious health risk for some time. In the longer-term, if pollutant concentrations fall by the 2050s as projected by the RCPs, then the health burden of NO_2_ will likely decrease on this timescale. The present study can help inform air quality emission control policies in order to benefit the population as a whole, as it is clear that air pollution will remain a major public health problem for some considerable time to come.

## Additional files

Additional file 1 Supplementary data analysis. This file contains additional data description, numerical and graphical summaries, and analysis not presented in the main paper. (PDF 1010 kb)

Additional file 2 Study data. This is a .zip file containing the data used in the study. The file also contains a read-me document summarising the content of the file. (ZIP 5070 kb)

## Abbreviations

AQUM: Air quality forecasting and modelling in the Unified Model
AURN: Automatic Urban and Rural Network
DEFRA: Department for the Environment, Food and Rural Affairs
EU: European Union
GMRF: Gaussian Markov Random Field
ICD-10: International Classification of Disease 10th Revision
IPCC: Intergovernmental Panel on Climate Change
JSA: Job Seekers Allowance LUA: Local and Unitary Authority
*m**a**x*_*s*_.*m**a**x*_*t*_: spatial max of the temporal max
*m**a**x*_*s*_.*m**e**a**n*_*t*_: spatial max of the temporal mean
MCMC: Markov chain Monte Carlo *m* *e* *a* *n*_*s*_.*m* *a* *x*_*t*_: spatial mean of the temporal max
*m**e**a**n*_*s*_.*m**e**a**n*_*t*_: spatial mean of the temporal mean
MetUM: Met Office Unified Model
MPP: Median Property Price
NO_2_: Nitrogen dioxide
NO _*x*_: Nitrogen Oxide
O_3_: Ozone
PM_2.5_: Particulate matter with an aerodynamic diameter less than 2.5 micrometers
PM_10_: Particulate matter with an aerodynamic diameter less than 10 micrometers
RCP: Representative Concentration Pathway
RR: Relative risk
SI: Sea ice
SMR: Standardised morbidity ratio
SO_2_: Sulphur dioxide
SSTs: Sea surface temperatures
UK: United Kingdom
WHO: World Health Organisation
