# Supplementary material for: Quantifying the impact of current and future concentrations of air pollutants on respiratory disease risk in England
Source: Environ Health. 2017 Mar 27;16:29. doi: 10.1186/s12940-017-0237-1 (PMC5368918; doi:10.1186/s12940-017-0237-1)

Additional file 1 for 'Quantifying the impact of current and future concentrations of air pollutants on respiratory disease risk in England'

Francesca Pannullo, Duncan Lee, Lucy Neal, Mohit Dalvi, Paul Agnew, Fiona M O'Connor, Sabyasachi Mukhopadhyay, Sujit Sahu, Christophe Sarran

Introduction

This file contains additional data description, numerical and graphical summaries, and analysis not presented in the main paper.

Exploratory analysis of the present day data

A map of the England study region is presented in Figure 1 (shaded region), where the locations of a number of the major cities are marked. One omission is the city of Birmingham that is referenced in the main paper, whose location is at the first 'N' in the word 'England'. A summary of the present-day disease (both raw hospital admission counts and the seasonally-adjusted SMR), pollution (using the $mean_s.mean_t$ metric), and covariate data is provided in Table 1, which presents the mean and standard deviation for each variable for each year. The table shows almost no change over the five year period in disease risk as measured by the seasonally-adjusted SMR, with the yearly means and standard deviations being almost identical. The increase over time in the raw disease counts is due to increasing populations in each LUA. Concentrations of NO_2, O_3 and PM_{10} show no evidence of trends, while slightly increasing and decreasing trends are observed for $PM_{2.5}$ and SO_2 respectively. Yearly average temperature levels are largely unchanged over the five year period, while JSA and MPP show increasing and decreasing trends respectively due to the global financial crisis that began in 2008 resulting in decreased economic activity.

The pollution data summarised above relate to the $mean_s.mean_t$ metric, and Figure 2 shows scatter plots of the relationships between the four different NO_2 metrics. The figure shows that there are strong positive relationships between the four aggregation metrics, which is also evidenced by the pairwise correlations that range between 0.929

Figure 1: Map displaying the geographical region of England, UK (shaded region).

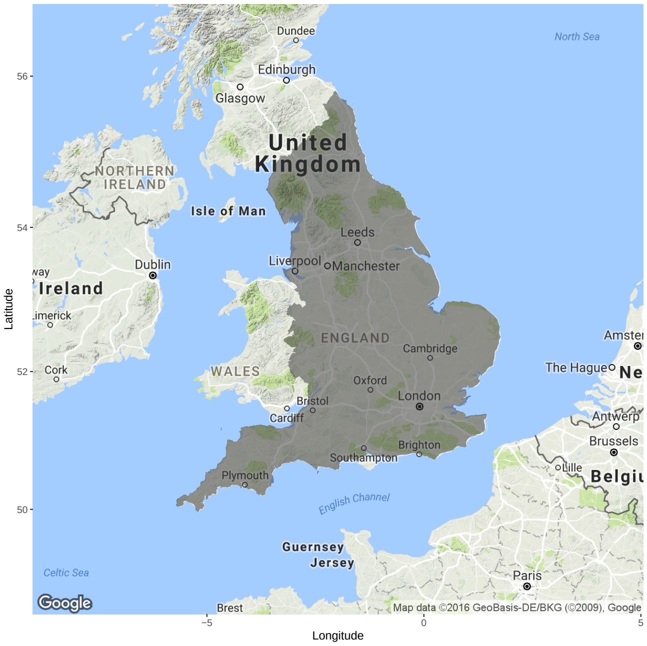

and 0.970. The pairwise correlations between the 4 aggregation metrics for the other pollutants range between 0.904 and 0.997, indicating high pairwise correlations between the metrics for each pollutant. In contrast, the between pollutant pairwise correlations for the $mean_s.mean_t$ metrics are displayed in Figure 3, which shows the strongest correlation of 0.803 between PM_{10} and $PM_{2.5}$ as expected. The correlation between O_3 and the remaining pollutants is negative and ranges between -0.68 to -0.083, suggesting it has its highest concentrations where the other pollutants have their lowest concentrations.

Exploratory analysis of the future pollution data

Table 2 displays the annual means and standard deviations (in brackets) in the 2050s for the future projected concentrations of pollutants separately for the three RCPs. The table relates to the $mean_s.max_t$ metric in all cases. The table shows that for NO_2,

Table 1: Mean and standard deviation (in brackets) of all the variables in the study for each year. SMR refers to the Standardised Morbidity Ratio, JSA refers to Job Seekers Allowance, MPP refers to Median Property Price.

Variable	2007	2008	2009	2010	2011
Admissions	146.5(132.4)	155.0(142.5)	158.7(142.6)	164.9(152.7)	165.8(144.8)
SMR	1.00(0.6)	1.00(0.6)	1.00(0.6)	1.01(0.6)	1.01(0.6)
NO_2 (μgm^{-3})	20.2(10.1)	19.5(9.2)	19.7(9.5)	21.1(10.2)	18.8(8.4)
O_3 (μgm^{-3})	47.1(13.2)	50.2(15.3)	47.2(13.7)	44.9(13.0)	48.0(11.8)
PM_{10} (μgm^{-3})	21.2(4.9)	18.0(4.6)	18.0(4.4)	18.8(3.8)	19.3(6.7)
$PM_{2.5}$ (μgm^{-3})	10.4(2.8)	10.8(2.7)	12.1(3.9)	13.0(3.6)	14.1(6.0)
SO_2 (μgm^{-3})	3.3(1.6)	3.3(1.6)	2.7(1.3)	2.7(1.6)	2.3(1.5)
JSA (%)	11.8(4.3)	12.6(4.5)	14.3(4.7)	13.9(4.6)	15.4(74.1)
MPP (£000)	191.1(66.0)	187.2(68.4)	178.4(65.5)	192.7(77.1)	189.3(79.5)
Temperature (°C)	10.5(3.9)	10.0(4.5)	10.2(4.8)	9.1(5.6)	11.0(4.1)

PM_{10}, $PM_{2.5}$ and SO_2 the highest concentrations come from RCP 6.0, while the lowest concentrations come from RCP8.5. The opposite pattern is again observed for O_3, which is due to the negative correlation between this pollutant and the others. NO_2 exhibited the strongest association with respiratory admissions and Table 3 presents the estimated future health impact of future NO_2 concentrations across all aggregation metrics. The $mean_s.max_t$ metric exhibited the greatest projected reduction in future respiratory admissions compared to the other metrics, with the $max_s.max_t$ metric showing similar reductions albeit not as strong. However, the $mean_s.mean_t$ metric shows a very slight projected increase in future admissions for RCP 2.6 and 6.0, while projecting a decrease under RCP8.5. Furthermore, the $max_s.mean_t$ metric projects a small admission increase for RCP 2.6, but not for the other two RCPs. Overall, NO_2 concentrations decrease across all 3 RCPs, however, the relative risk was highest for the $mean_s.mean_t$, which is indicative of the project slight increases shown here. It is worth noting that the two metrics which project an increase in admissions are the two metrics which utilise a temporal monthly average, rather than a temporal monthly maximum. Again, this highlights that it is extremely important to take into account different aggregating metrics since they can produce different results.

Residual spatial structure in the disease data

As described in the main paper, the spatio-temporal pattern in disease risk is modelled by known covariates and a set of random effects, the latter accounting for factors such as unmeasured confounding. The spatio-temporal autocorrelation in these effects is accounted for in the modelling process, and the common approach assumes this unmeasured structure has a single level of spatial smoothness across the study region.

Figure 2: Scatterplots of the relationships between the four aggregated NO_2 metrics, namely $mean_s.mean_t$, $max_s.mean_t$, $mean_s.max_t$, $max_s.max_t$, along with density plots and pairwise correlations.

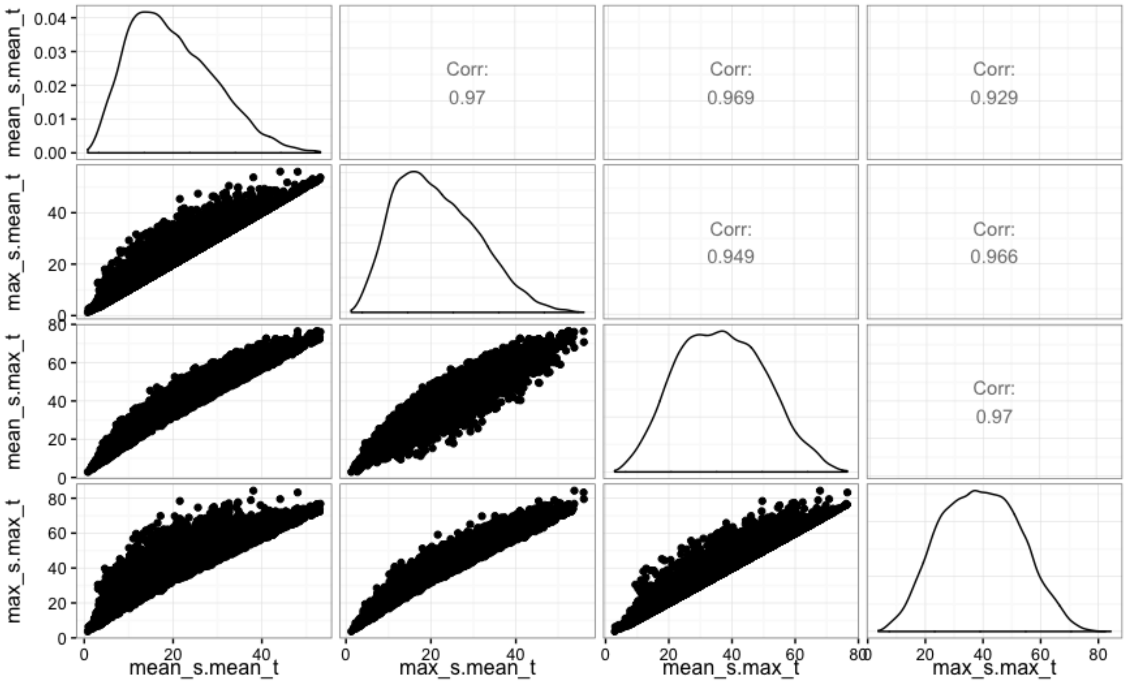

Figure 4 presents residuals on the natural log scale (averaged over all $T = 60$ months) from a simpler model without the random effects, and shows that the spatial pattern does not exhibit a globally smooth pattern. Instead, some pairs of adjacent LUA have similar residual values suggesting spatial autocorrelation, while between other pairs there are large step changes. This motivates the use of the localised spatial smoothing model used in the paper, which allows for this more nuanced and localised residual autocorrelation structure.

Table 2: Mean and standard deviation (in brackets) of the future projected concentrations of pollutants (using the $mean_s.max_t$ metric) for each year and RCP. All pollutants are measured in μgm^{-3} across the 2050s.

RCP	Pollutant	Year 1	Year 2	Year 3	Year 4	Year 5
2.6	NO_2	21.6(8.1)	21.5(8.6)	21.3(7.5)	21.3(7.8)	23.0(9.4)
	O_3	60.0(10.9)	60.5(11.7)	59.0(10.7)	61.4(10.3)	60.4(12.3)
	PM_{10}	19.8(6.3)	17.8(4.6)	19.7(6.9)	19.1(6.6)	21.1(7.5)
	$PM_{2.5}$	11.7(5.0)	10.2(3.5)	10.9(3.1)	11.6(6.3)	11.8(4.3)
	SO_2	1.5(0.7)	1.4(0.7)	1.4(0.7)	1.5(0.6)	1.7(0.8)
6.0	NO_2	23.0(10.1)	22.1(8.2)	25.0(10.9)	21.0(8.9)	23.4(9.4)
	O_3	66.1(13.6)	68.2(13.3)	68.5(17.5)	70.8(14.1)	71.0(15.8)
	PM_{10}	23.5(4.5)	25.2(5.5)	27.4(9.5)	22.9(5.2)	25.0(8.1)
	$PM_{2.5}$	15.1(4.1)	16.7(4.8)	19.1(8.3)	15.2(4.8)	16.6(6.2)
	SO_2	4.5(1.9)	4.0(1.7)	4.8(2.4)	4.0(1.7)	4.4(1.6)
8.5	NO_2	14.1(5.4)	14.7(5.7)	15.1(6.5)	14.5(5.8)	14.4(5.7)
	O_3	78.9(20.3)	80.2(20.3)	76.4(20.1)	80.8(19.2)	81.0(17.0)
	PM_{10}	17.3(4.1)	18.6(5.0)	17.8(5.5)	20.8(9.4)	22.1(12.1)
	$PM_{2.5}$	9.8(3.6)	10.9(4.5)	9.9(4.1)	12.1(7.7)	13.6(9.9)
	SO_2	0.4(0.5)	0.4(0.4)	0.5(0.5)	0.5(0.5)	0.5(0.5)

Table 3: Estimated change in future respiratory hospital admissions per year and 95% credible intervals in brackets for all NO_2 aggregation metrics. $\uparrow$ represents an increase in future admissions, whereas a $\downarrow$ represents a reduction in future admissions.

RCP	$\text{mean}_s.\text{mean}_s$	$\text{mean}_s.\text{max}_t$	$\text{max}_s.\text{mean}_t$	$\text{max}_s.\text{max}_t$
2.6	$\uparrow$ 2,291 (993, 3,965)	$\downarrow$ 10,487 (15,435, 4,637)	$\uparrow$ 643 ($\downarrow$ 33, 1,364)	$\downarrow$ 8,601 (13,908, 3,169)
6.0	$\uparrow$ 4,027 (1,760, 6,904)	$\downarrow$ 8,659 (12,740, 3,615)	$\downarrow$ 192 (283,$\uparrow$ 13)	$\downarrow$ 7,205 (11,639, 2,657)
8.5	$\downarrow$ 6,123 (10,066, 2,772)	$\downarrow$ 14,661 (21,546, 6,128)	$\downarrow$ 5,053 (10,078, $\uparrow$ 274)	$\downarrow$ 8,037 (12,984, 2,963)

Figure 3: Scatterplots of the relationships between the 5 pollutants considered in this study, namely NO_2, O_3, PM_{10}, $PM_{2.5}$ and SO_2. In all cases the $mean_s.mean_t$ aggregation metric is presented.

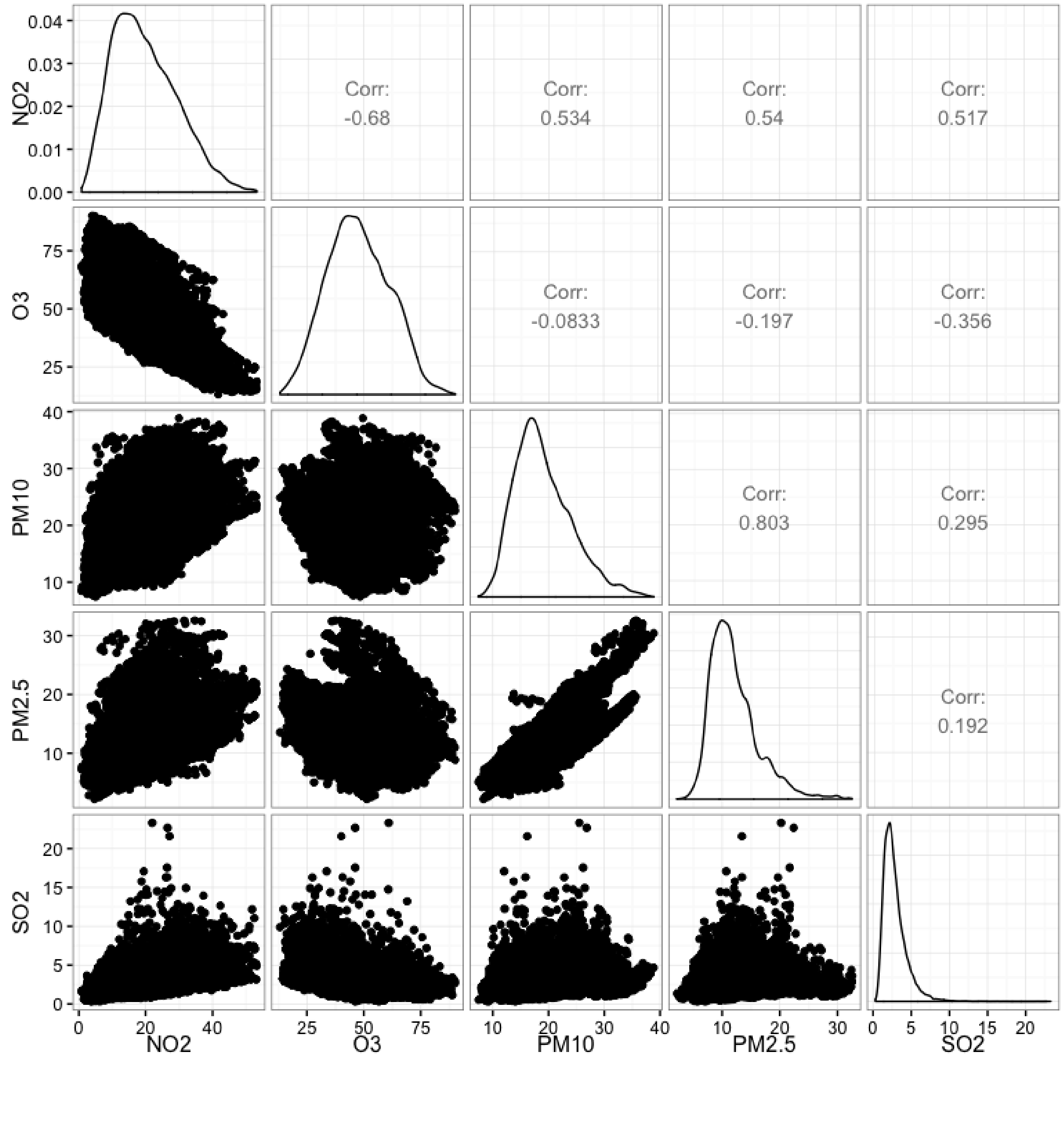

Figure 4: Map displaying the residuals on the natural log scale (averaged over all $T = 60$ months) from a simple model with only the covariates, which displays the unexplained spatial pattern in the disease data.

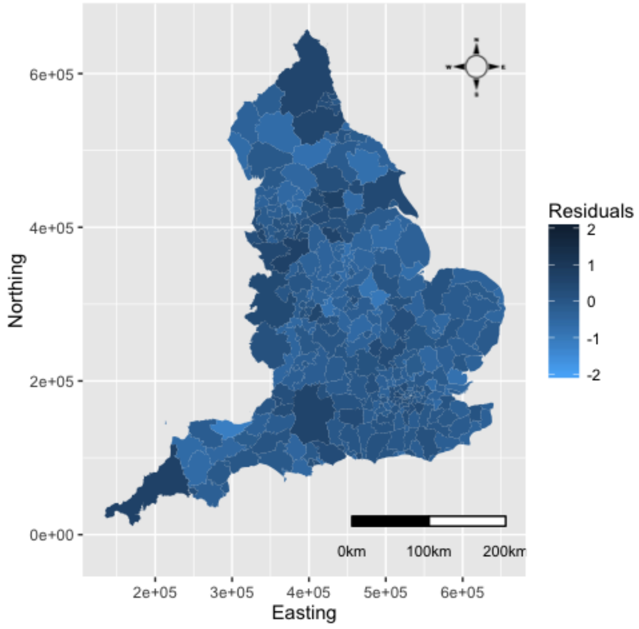

Supplement: Supplementary file 1 — Supplementary data analysis. This file contains additional data description, numerical and graphical summaries, and analysis not presented in the main paper. (PDF 1010 kb) [file 12940_2017_237_MOESM1_ESM.pdf]
