# Supplementary material for: Quantifying the impact of current and future concentrations of air pollutants on respiratory disease risk in England
Source: Environ Health. 2017 Mar 27;16:29. doi: 10.1186/s12940-017-0237-1 (PMC5368918; doi:10.1186/s12940-017-0237-1)
Supplement: Supplementary file 2 — Study data. This is a .zip file containing the data used in the study. The file also contains a read-me document summarising the content of the file. (ZIP 5070 kb) [file 12940_2017_237_MOESM2_ESM.zip › Data/README.docx]

# README

## Present-day Data

**Current_data.csv** holds the present-day air pollutant concentrations and disease data along with weather and deprivation data.

- LAD11CDO, LAD11CD are the LUA codes.
- Time_period, month and year refer to the study period where time_period is a combination of month and year.
- Resp_o and resp_expected refer to the observed and expected counts of disease.
- Ozone.mean.mean, ozone.max.mean, ozone.mean.max, ozone.max.max (similar names for all pollutants) refers to the four spatio-temporal aggregation metrics (spatial mean of the temporal mean, spatial maximum of the temporal mean, spatial mean of the temporal maximum, and spatial maximum of the temporal maximum). All measured in µgm^-3^.
- Temperature is measured in kelvin.
- JSA (Job Seekers Allowance), MPP (Median Property Price) refer to the socio-economic data where JSA is a percentage and MPP is in pounds.
- EnewR refers to the seasonally-adjusted SMR (Standardised Morbidity Ratio).

## Future pollutant Data

These data files contain the temporal maximum spatial mean/maximum future pollutant data for each of the three RCPs (2.6, 6.0, 8.5). Each file contains the LUA codes, study period codes and each of the 5 pollutants (measured in µgm^-3^).
